# Extended Heterosubtypic Neutralization and Preclinical Model In Vivo Protection from Clade 2.3.4.4b H5 Influenza Virus Infection by Broadly Neutralizing Antibodies

**DOI:** 10.3390/vaccines14010071

**Published:** 2026-01-08

**Authors:** Valeria Caputo, Martina Libera, Yailin Campos Mota, Kaito Nagashima, Ana Maria Moreno Martin, Claudia Maria Trombetta, Francesca Dapporto, Jarrod J. Mousa, Emanuele Montomoli, Giuseppe A. Sautto, Roberta Antonia Diotti

**Affiliations:** 1 Pomona Ricerca S.r.l., 10122 Turin, Italy; caputo.valeria@pomonaricerca.com (V.C.); libera.martina@pomonaricerca.com (M.L.); 2 Florida Research and Innovation Center, Cleveland Clinic, Port Saint Lucie, FL 34987, USA; 3 Department of Infectious Diseases, Center for Vaccines and Immunology, College of Veterinary Medicine, University of Georgia, Athens, GA 30602, USA; kan4536@caltech.edu (K.N.);; 4 Virology Department, Istituto Zooprofilattico Sperimentale della Lombardia e dell’Emilia-Romagna, 25124 Brescia, Italy; anamaria.morenomartin@izsler.it; 5 Department of Molecular and Developmental Medicine, University of Siena, 53100 Siena, Italy; 6 VisMederi S.r.l, 53100 Siena, Italy; 7 Department of Biomedical Sciences, College of Medicine, Florida State University, Tallahassee, FL 32306, USA

**Keywords:** influenza virus, one health, monoclonal antibodies, immunotherapy, avian influenza virus

## Abstract

**Background/Objective**: The influenza virus remains one of the most prevalent respiratory pathogens, posing significant global health and economic challenges. According to the World Health Organization, the seasonal influenza virus infects up to 1 billion people and causes up to 650,000 deaths, annually. Despite influenza vaccination is the most effective available preventive strategy, its reliance on strain predictions and yearly updates limits its effectiveness. The virus’ ability to cause both epidemics and pandemics, driven by zoonotic transmissions, underscores its continuous threat. The ongoing H5N1 avian influenza outbreak is the perfect example, renewing concerns due to its ability to infect over 70 mammalian species and sporadically transmit to humans. This study aims to evaluate the protective potential of two human monoclonal antibodies against diverse and recent influenza virus strains. **Method**: PN-SIA28 and PN-SIA49 monoclonal antibodies were previously isolated from an individual undergoing seasonal influenza vaccination and with no known recent influenza virus exposure. Their breadth of recognition, neutralization, and conferred in vivo protection were assessed against multiple influenza viruses, including pre-pandemic strains. Structural analyses were performed to characterize antibody–antigen interactions for epitope identification. **Results**: Both antibodies recognize a broad range of strains and neutralize pre-pandemic avian influenza viruses, including the currently circulating H5N1 clade. Moreover, a structural analysis revealed that PN-SIA49 binds a conserved HA stem region, overlapping with epitopes recognized by other broadly neutralizing antibodies. **Conclusions**: These findings underscore the potential of broadly neutralizing antibodies as a basis for universal influenza countermeasures against both seasonal and pandemic threats. Additionally, they provide guidance for the design of targeted vaccine strategies to steer immune responses toward broadly protective epitopes.

## 1. Introduction

The influenza virus is one of the most prevalent respiratory pathogens significantly impacting the world healthcare and economic systems each year [1]. According to the World Health Organization (WHO), up to 1 billion people are infected annually by the seasonal influenza virus, with 3 to 5 million experiencing severe diseases culminating in 290,000–650,000 deaths due to respiratory complications [2]. Until now, the most effective strategy to prevent influenza and to reduce symptoms is represented by the annual seasonal vaccination, which is recommended for all groups, especially high-risk populations [3]. Currently, the seasonal influenza virus vaccine is composed of two influenza A virus subtype strains, H1N1 and H3N2, and one strain of the influenza B virus, belonging to the Victoria lineage, that are annually selected by the WHO.

Specifically, the WHO’s Global Influenza Surveillance and Response System (GISRS) monitors the circulating strains and recommends the specific virus isolates that should be included in the upcoming influenza vaccine [4]. However, this vaccine approach needs annual updates as it relies on predicting which influenza virus strain will be the most prevalent during the upcoming winter season. These predictions are made several months in advance by leveraging global surveillance data. However, given the influenza virus’ high mutation rate and the possible emergence of new variants, the vaccine strains may not always match the circulating viruses, which can significantly reduce the vaccine effectiveness. Even when the vaccine strains are antigenically well-matched with the circulating viruses, the vaccine effectiveness ranges between 40–60% [5]. Moreover, in the case of influenza vaccines produced using fertilized chicken eggs, the efficacy can also be affected by egg-adaptation mutations and glycosylation. This is more common for H3N2 influenza strains that grow poorly in eggs [6] and may undergo adaptative mutations, which can alter the antigenic properties of the vaccine strain, with a consequent possible mismatch with the circulating strains and a reduction in the overall protective efficacy [7].

Two main mechanisms are responsible for the influenza virus evolution, antigenic drift and antigenic shift, especially at the level of the main viral surface glycoproteins, hemagglutinin (HA), and/or neuraminidase (NA). While antigenic drift is the progressive accumulation of point mutations in circulating viruses that can cause seasonal epidemics, antigenic shift results in a drastic change in the virus surface glycoproteins [8]. These novel antigens can emerge via the reassortment of animal- and human-derived strains in an intermediate host, like pigs. This leads to the emergence of entirely new subtypes previously not circulating in the human population and thus not recognized by the human immune system and with the potential of causing a pandemic [5].

There is a growing global concern over avian influenza virus outbreaks and the increasing risk of zoonotic transmission, especially of H5N1 strains belonging to the clade 2.3.4.4b [9,10]. Viruses of this clade were first identified in Europe in October 2020, following the reassortment between the H5N8 and N1 viruses of wild avian origin. Since 2021, they have become globally dominant, exhibiting efficient geographical dissemination via migratory birds. In recent years, numerous H5 highly pathogenic avian influenza virus (HPAIV) outbreaks have occurred in wild birds and domestic poultry worldwide, except in the Oceania continent. Since then, the virus has also crossed species barriers, infecting at least 70 mammalian species, further increasing the risk of adaptation to humans. In the past two years, the Centers for Disease Control and Prevention (CDC) has documented 100 human infections (71 cases in 2024 and 29 in 2025) [11]; however, no instances of human-to-human transmission have been reported [8]. Moreover, since 2024, infections have been detected in lactating dairy cows in the United States, with 1081 outbreaks reported across 18 states and high viral loads detected in milk, raising serious public health concerns [12]. Considering the potential catastrophic consequences of viral adaptations on public health, agriculture, and biodiversity, it is imperative to explore new strategies for preparedness and response.

In the last century, four major pandemics have been reported, including the most recent swine-origin flu (H1N1) pandemic of 2009–2010 [13].

Our group has previously described two human monoclonal antibodies (mAbs), PN-SIA28 and PN-SIA49, both endowed with a broad biological activity against multiple influenza virus subtypes. Both antibodies were isolated from a patient with a negative clinical history of influenza infection in the last 10 years but with detectable and durable serum antibody neutralizing titers derived from previous exposures and seasonal vaccination [14]. Previous evaluations of the neutralizing activity of these two antibodies showed their broad neutralization of influenza viruses belonging to group 1 [15], with PN-SIA28 also neutralizing influenza viruses belonging to group 2 [9,16]. In more detail, PN-SIA28 neutralized H3N2 strains isolated between 1968 and 1975 [16].

Additionally, these antibodies demonstrated therapeutic efficacy against HPAIV H5Nx influenza strains belonging to the clades 2.3.4.4 and 1 such as H5N6 A/Shenzhen/TH002/2016 (PN-SIA28) and H5N1 A/Vietnam/1203/2004 (PN-SIA49) [9,15,17]. In both cases, the avian viral strains caused isolated human infections that also led to one death [9,18].

In this work, we evaluated the extent of protection conferred by PN-SIA28 and PN-SIA49 against emerging influenza strains and assessed their breadth of recognition across a wide range of HA glycoproteins, including recent influenza viruses from different subtypes. Our findings highlight the presence of broad-spectrum influenza antibodies elicited by previous influenza virus infections or vaccinations and endowed with heterosubtypic biological activity, providing guidance for influenza vaccine improvement strategies.

## 2. Material and Method

### 2.1. Monoclonal Antibodies

The monoclonal antibodies PN-SIA49 and PN-SIA28 are human IgG1 antibodies. PN-SIA49 binds and neutralizes group 1 influenza A viruses, while PN-SIA28 binds and neutralizes both group 1 and group 2 influenza A viruses. Both antibodies were purified from transfected Chinese hamster ovary (CHO) cells using a one-step purification with HiTrap^TM^ Protein A columns by Genscript (Nanjing, China).

Plasmid sets for the IgG1 isotype control anti-SARS coronavirus human mAb CR3022 (NR-53260) were produced under HHSN272201400008C and obtained through BEI Resources, National Institute of Allergy and Infectious Disease (NIAID), and National Institution of Health (NIH) [19]. The mAb was purified from the conditioned supernatants of transfected cells and quantified, as previously described [20].

### 2.2. Cell Line

Madin-Darby Canine Kidney (MDCK) cells (cat. n. CCL-34, American Type Culture Collection [ATCC], Manassas, VA, USA) were maintained in Dulbecco’s Modified Eagle Medium (DMEM) supplemented with penicillin–streptomycin (PenStrep) (cat. n. 15070063, Thermo Fisher Scientific, Waltham, MA, USA), 25 mM HEPES buffer (cat. n. 15630080, Thermo Fisher Scientific), and 10% heat-inactivated fetal bovine serum (FBS) (cat. n. ECS5000L, Euroclone, Pero, Italy).

For the production and titration of H5 pseudotyped virus (PV), human embryonic kidney 293 T/17 (HEK293 T/17) (cat. n. CRL-11268, ATCC) were maintained in DMEM high glucose, pyruvate (cat. n. 11995073, Gibco, Thermo Fisher Scientific) supplemented with 10% of FBS and 1% PenStrep (cat. n. P4333, Merck, Rahway, NJ, USA). Cells were cultured at 37 °C, 5% CO_2_.

### 2.3. Viruses

H1N1 A/Brisbane/02/2018 (Brisb/18), H3N2 A/Hong Kong/4801/2014 (HK/14), and H5N6 A/Sichuan/26221/2014 (Sich/14) influenza strains were obtained through the Influenza Reagents Resource (IRR), BEI Resources, or the CDC. All viruses were passaged once under the same growth conditions as those in which they were received, using 10-day-old embryonated specific pathogen-free (SPF) chicken eggs (cat. n. 10100326, AVSBio, Norwich, CT, USA), following the protocol recommended by the WHO [21].

H5N1 HPAIV clade 2.3.4.4b A/duck/Italy/326224/2/22VIR909/2022 (Duck/22) was obtained from Istituto Zooprofilattico Sperimentale della Lombardia e Dell’Emilia Romagna (IZSLER, Brescia, Italy). The strain was propagated in MDCK cells as previously described [22].

### 2.4. rHA Proteins

All the recombinant HA (rHA) proteins described below were expressed in EXPI293 cells. C-terminal histidine tag with HisTrap excel nickel-affinity chromatography columns (cat. n. 17371205, Cytiva, Marlborough, MA, USA) were used to purify the protein as previously described [23] and then dialyzed against phosphate-buffered saline (PBS) (cat. n. 21-040-CM, Corning, Corning, NY, USA). Protein concentration was quantified by bicinchoninic acid (BCA) assay (cat. n. 23227, Thermo Fisher Scientific) and adjusted to ~1 mg/mL. Sodium dodecyl sulfate-polyacrylamide gel electrophoresis (SDS-PAGE) and Western blot (Thermo Fisher Scientific) were performed to test the purity of our preparation.

For binding assays, the full-length ectodomain of HA proteins was developed for a number of recent and historical vaccine or wild-type components for H1N1 (A/Singapore/6/1986 (Sing/86), A/New Caledonia/20/1999 (NC/99), A/Brisbane/59/2007 (Brisb/07), A/California/04/2009 (CA/09), A/Michigan/45/2015 (Mich/15), A/Brisbane/02/2018 (Brisb/18), A/Guangdong-Maonan/SWL1536/2019 GM(19), A/Wisconsin/588/2019 (Wisc/19), A/Victoria/4897/2022 (Vic/22), G4 [24], H3N2 (A/Port Chalmers/1/1973 (PC/73), A/Panama/2007/1999 (Pan/99), A/Victoria/361/2011 (Vic/11), A/Texas/50/2012 (TX/12), A/Switzerland/9715293/2013 (Switz/13), A/Hong Kong/4801/2014 (HK/14), A/Singapore/INFIMH-16 0019/2016 (Sing/16), A/Switzerland/8060/2017 (Switz/17), A/South Australia/34/2019 (SA/19), A/Hong Kong/2671/2019 (HK/19), A/Tasmania/503/2020 (Tas/20) and H5N1 A/Vietnam/1203/2004 (Viet/04), A/whooper swan/Mongolia/244/2005 (WS/05), A/chicken/Egypt/CAL3-RLQP/2017 (ChickEgypt/17), A/dairy cattle/Texas/24-008749-002/2024 (TX/24), H5N6 A/Sichuan/26221/2014 (Sich/14), and H5N8 A/gyrfalcon/Washington/41088-6/2014 (Gyrfalc/14). The rHA of the Computationally Optimized Broadly Reactive Antigen (COBRA) HA Y2 [21] was used for the structural studies involving PN-SIA49.

### 2.5. Binding Kinetics and Activities of PN-SIA49 and PN-SIA28

Binding kinetics of PN-SIA28 and PN-SIA49 mAbs to a panel of rHA proteins was determined by biolayer interferometry (BLI) using an Octet RH16 system and Anti-Penta-HIS (HIS1K) biosensors (Sartorius, Göttingen, Germany). rHAs were used at a final concentration of 50 μg/mL. Prior to analysis, biosensors were hydrated for 10 min in kinetic buffer (1× PBS + 0.5% bovine serum albumin [BSA]). MAb and rHA dilutions were prepared in the same buffer, which was also used for baseline and dissociation steps. MAb binding affinity was assessed using an automated four-step procedure composed of a 60 s reference baseline, a 200 s loading phase, and a 300 s association (k_on_) using seven 2-fold mAb dilutions (133.3, 66.7, 33.3, 16.7, 8.33, 4.17, and 2.08 nM). Dissociation (k_off_) was then performed for 600 s. All steps were performed at 25 °C with an agitation of 1000 rpm. Data analysis was performed using Data Analysis Studio (version 12.2), and mAb Kd calculated as the ratio of k_off_ to k_on_ was determined using a 1:2 interaction model.

For the binding studies to H5 belonging to clade 2.3.4.4b, pre-formed monolayer of MDCK cells (90%) was infected with 100 50% Tissue Culture Infectious Dose (TCID_50_) of Duck/22 H5N1 HPAIV and incubated at 37 °C for 3 days. The infected cells were then fixed with 4% paraformaldehyde for 30min at room temperature (RT), followed by three washes with PBS. PN-SIA28 and PN-SIA49 were then added for 1 h. The mAb binding was detected using a horseradish peroxidase (HRP)-conjugated anti-human antibody (cat. n. A0293, Merck). A mouse mAb specific for influenza A virus nucleoprotein A (HB65, H16-L10-4R5, ATCC) was used as a positive control, followed by HRP-conjugated anti-mouse antibody (cod. 72689, IZSLER).

### 2.6. Pseudotyped Virus Production

The p8.91 lentiviral package expressing gag-pol from HIV and pCSFLW containing firefly luciferase reporter gene were kindly provided by Prof. Nigel Temperton (Viral Pseudotype Unit, Medway School of Pharmacy, University of Kent, Chatham Maritime, UK). The full-length HA gene from H5N1 TX/24 (clade 2.3.4.4b) was synthesized and subcloned into pTR600 by Azenta Life Science (Burlington, MA, USA).

Twenty-four hours before the transfection, 4 × 10^6^ HEK293 T/17 were seeded in p100 and incubated at 37 °C, 5% CO_2_ overnight. The next day, the medium was replaced, and cells were transfected with the plasmid DNA mixture consisting of 500 µL Opti-MEM 1X reduced serum medium (cat. n. 31985-070, Gibco, Thermo Fisher Scientific) with 260 ng p8.91, 375 ng pCSFLW, 250 ng H5, and 2.66 µL of EndoFectin^TM^ -Lenti (cat. n. EF001, GeneCopoeia, Rockville, MD, USA). Twenty-four hours post-transfection, 1 U neuraminidase (cat. n. N2876, Sigma-Aldrich, St. Louis, MO, USA) was added to the plates. At 72 h post-transfection, PV particles were collected, centrifuged, filtered through a 0.45 µm filter syringe, and stored at −80 °C.

### 2.7. Pseudotyped Virus Titration

The titration was performed in a white 96-well plate format. Viral supernatants were serially diluted two-fold in duplicate, and 50 µL of 2 × 10^6^ HEK293 T/17 cell/mL were added. A negative control (cells only) was also included on each plate. Plates were then incubated at 37 °C, 5% CO_2_ for 72 h. Following incubation, 50 µL of Bright-Glo Luciferase Assay System (cat. n. E2620, Promega, Madison, WI, USA) were added to each well, and the plate was shaken for 5 min at 400 rpm in the dark. Luminescence was measured using a luminometer (GloMax, Promega), and the PV titer, expressed as relative luminescence units per milliliter (RLU/mL), was calculated using Excel.

### 2.8. PN-SIA49 and PN-SIA28 Neutralization Assays

*Neutralization assay against HK/14*. On the day of the assay, each well of a 96-well flat-bottom microtiter plate (Thermo Fisher Scientific) was filled with 50 μL of virus diluent DMEM, 1% PenStrep, 13.5% BSA, 2.5% 1 M HEPES buffer, 2 μg/mL l-(tosylamido-2-phenyl) ethyl chloromethyl ketone (TPCK) trypsin (cat. n. 20233, Thermo Fisher Scientific). Forty μL of virus diluent was then added, followed by 10 μL of PN-SIA49 and PN-SIA28 at a concentration of 200 μg/mL. Two-fold serial dilutions were established by transferring 50 μL from the first column sequentially across the plate, with the final 50 μL discarded at column 10. Subsequently, 50 μL of HK/14 virus, previously titrated to 100× of the 50% tissue culture infectious dose (100× TCID50), was added to each well, except those in column 12. The plates were then incubated at 37 °C for 1 h, after which 100 μL of 1.5–2 × 10^5^ cells/mL MDCK cells were added. The plates were then incubated at 37 °C + 5% CO_2_ for 18–20 h. The next day, each well was washed twice with 200 μL of PBS and fixed with ice-cold fixative solution (80% Acetone (Thermo Fisher Scientific)) diluted in PBS for 10 min at RT. The fixative solution was then wasted, and the plates let dry for 1 h. The plates were then washed three times with 200 μL wash buffer (PBS + 0.03% Tween-20 (Thermo Fisher Scientific)). The plates were then decanted, and 100 μL of rabbit anti-influenza-A-NP polyclonal antibody (1 mg/mL, cat. n. PA5-81661, Thermo Fisher Scientific) diluted 1:3000 in blocking buffer (PBS + 0.03% Tween-20 + 14.5% BSA) was added to each well. The plates were then incubated in a humidified chamber at RT for 1 h and then washed three times with 200 μL wash buffer. A total of 100 μL of goat-anti-rabbit IgG (H + L) HRP (1 mg/mL, cat. n. 31460, Thermo Fisher Scientific) diluted 1:5000 in blocking buffer was added to each well. The secondary antibody was then incubated in a humidified chamber at RT for 1 h. The plates were then washed five times with 200 μL wash buffer, and 100 μL of the substrate (phosphate citrate buffer (Sigma-Aldrich), o-phenylenediamine dihydrochloride (OPD) tablets (Sigma-Aldrich), 0.05% 30% H_2_O_2_) were added to each well. The reaction was allowed to develop for 3–5 min at RT and stopped by adding 50 μL of 2N H_2_SO_4_ (Thermo Fisher Scientific). The plates were then read at 492 nm using a BioTek Epoch 2 plate reader (Agilent, Santa Clara, CA, USA, Gen6 software version 1.03). The 50% neutralization titer was calculated by subtracting the background signal from the mean optical density (OD) of the virus control wells and defining this value as 100% infection. The OD values from experimental wells were then normalized to the 100% infection control, and a 50% inhibition concentration (IC_50_) for each mAb was calculated using a non-linear regression interpolation of the values from each mAb dilution. All mAb dilutions were tested in duplicate and the average neutralization activity was determined.

*Neutralization assay against Duck/2022.* The virus microneutralization test (MNT) was performed in a BSL3 laboratory according to previously described procedures [25]. Briefly, 2-fold serial dilutions of the PN-SIA28 and PN-SIA49 starting from 132 and 128 μg/mL, respectively, were incubated for 1 h at 37 °C with an equal volume of 100 TCID_50_ of Duck/22 H5N1 HPAIV. After incubation, 50 µL of an MDCK cell suspension containing 1 × 10^5^ cells/mL in 10% fetal calf medium was added and the plate was incubated for 72 h at 37 °C with 5% CO_2_. After three days of incubation, cells were washed 3 times with PBS, fixed with 80% cold acetone, and subjected to an indirect immunoperoxidase assay performed as previously described [26]. The 100% inhibition concentration (IC_100_) was defined as the lowest mAb dilution that prevents viral infection of MDCK cells, determined by the complete absence of influenza virus infection as detected by the immunoperoxidase reaction.

*Pseudotyped virus-based microneutralization assay.* The pseudotyped virus-based microneutralization (pMN) assay was performed in a white 96-well plate format. Immunoglobulin-depleted serum (cat. n. SF505-2, BBI Solutions, Cardiff, UK) and A/Vietnam/1194/04 (NIBRG-14) were used as negative and positive controls, respectively. MAbs PN-SIA28 and PN-SIA49 were tested using a range concentration of 10–0.02 µg/mL. Ten microliters of mAbs were diluted in 90 µL of DMEM and a serial two-fold dilution was performed. Fifty microliters of a PV dose input equal to 1 × 10^6^ RLU/well, as determined via titration, was then added. PV-only (equivalent to 0% neutralization) and cell-only controls with no virus (equivalent to 100% neutralization control) were also included in the plates. Plates were incubated for 1 h at 37 °C, 5% CO_2_, and then 2 × 10^6^ HEK293 T/17 cell/mL were added to each plate. After 72 h, 50 µL of the Bright-Glo^®^ luciferase assay substrate (Promega) was added to each well. Plates were vortexed for 5 min at 400 rpm in the dark and read using a luminometer (Promega). RLU values were normalized relative to the PV only and cells only, using GraphPad Prism version 10. The resulting neutralization-antibody dose–response curve was modelled using a non-linear regression model (log (inhibitor) vs. normalized response–variable slope) analysis.

*Focus reduction assay*. The focus reduction assay (FRA) was performed as previously described [27]. Briefly, MDCK-SIAT1 cells were seeded at a concentration of 2.5–3 × 10^5^ cells/mL in culture medium in a 96-well plate (cat. n. 655101, Greiner Bio-One, Kremsmünster, Austria). The following day, cells were washed with PBS (Corning), and a 2-fold serial dilution of PN-SIA49 was prepared starting at 20 µg/mL in 50 µL virus growth medium (VGM) supplemented with 1 µg/mL TPCK-treated trypsin (cat. n. 20233, Thermo Fisher Scientific) was added in duplicate to the wells. Subsequently, 50 µL of virus Brisb/18 or Sich/14 at 1.2 × 10^4^ focus-forming units/mL were added to each well, including the positive control wells. The plate was incubated at 37 °C with 5% CO_2_; thereafter, 100 µL per well of overlay was added and the plate was incubated overnight (18–22 h) at 37 °C with 5% CO_2_. The following day, the overlays were removed, and the cell monolayers were washed once with PBS. The monolayers were ice-cold fixed with 4% formalin for 30 min at 4 °C, washed with PBS, and permeabilized with 0.5% Triton X-100 (cat. n. X100, Sigma-Aldrich) in PBS/glycine at RT for 20 min. The plate was washed three times with PBS supplemented with 0.1% Tween 20 and incubated with mAb against influenza A nucleoprotein (IRR) in ELISA buffer. After a 1 h incubation at 37 °C, the plate was washed three times, and secondary antibody (anti-mouse IgG, peroxidase labelled, cat. n. 474-1802, KPL, New Delhi, India) was added. Following a 1 h incubation at 37 °C, infectious foci were visualized using TrueBlue substrate (cat. n. 5510-0030, SeraCare, Milford, MA, USA) containing 0.03% H_2_O_2_ incubated at RT for 10–15 min. The reaction was stopped by washing five times with distilled water. Once the plate had dried, the foci were enumerated using an S6 Macro ELISPOT reader with ImmunoCapture 6.4.87 software (Cellular Technology Limited [CTL], Shaker Heights, OH, USA).

### 2.9. Mouse Infections: Prophylactic and Therapeutic Regimens

DBA/2J mice (females, 6–8 weeks old) were obtained from the Jackson Laboratory. Animals (Bar Harbor, ME, USA) were housed in microisolator units, provided food and water throughout the study, and maintained in accordance with USDA guidelines for laboratory animals’ care. All experimental procedures were approved by the University of Georgia Institutional Animal Care and Use Committee (IACUC) (n. A2021-06-016-Y1-A2; initial approval date: 9 September 2021; renewal date: 9 September 2024). Mice were randomly divided into groups (8 animals/group) and either used for the prophylactic or therapeutic studies described below.

*Therapeutic study.* For this study, 24 h before infection, mice received a single intraperitoneal dose of PN-SIA49 (15 mg/kg), CR3022 (15 mg/kg), or PBS (control). Mice were inoculated intranasally, under isoflurane anesthesia, with 10^5^ PFU/mouse of Brisb/18, or 10^5^ PFU/mouse of Sich/14 influenza viruses. Mice body weight loss and mortality were monitored daily over a period of 14 days. Animals that lost more than 25% of body weight were euthanized. On day 3 post infection (p.i.), subgroups of 3 randomly selected mice were sacrificed for the determination of lung viral titers (LVT). The lungs were sampled aseptically and homogenized in DMEM supplemented with PenStrep, and supernatants were titrated by using standard plaque assays as described below.

*Prophylactic study.* The mice were inoculated intranasally, under isoflurane anesthesia, with 10^5^ PFU/mouse of Brisb/18, or 10^5^ PFU/mouse of Sich/14 influenza viruses. At 24 h p.i., infected animals received a single intraperitoneal dose of PN-SIA49 (10 mg/kg or 20 mg/kg), CR3022 (20 mg/kg), or PBS (control). Mice body weight loss and mortality were monitored over a period of 14 days. Animals that lost more than 25% of body weight were euthanized. Subgroups of 3 randomly selected mice were sacrificed on day 3 p.i. for determination of LVT. The lungs were sampled aseptically and homogenized in DMEM supplemented with PenStrep, and supernatants were titrated by using standard plaque assays as described below.

### 2.10. Lung Viral Titer Plaque Assay

The lung viral titers were assessed by plaque assay. The viral titers were tested in 24-well plates seeded with MDCK cells and incubated at 37 °C, 5% CO_2_. The following day, the wells were washed with fresh DMEM while preparing the serial dilutions to be used for viral titration: 10^−1^, 10^−2^, 10^−3^, and 10^−4^. The dilutions were made in DMEM + 1 μg/mL TPCK-treated trypsin (cat. n. 20233, Thermo Fisher Scientific) in duplicates and added to the plates. A negative control, composed of DMEM + TPCK only, was also added to the plates. The infection was left for 1 h at RT and gently rocked every 10 min. The plates were washed with fresh DMEM, after which the overlay medium (DMEM + 0.3% agarose (final concentration) + 1 μg/mL TPCK-treated trypsin) was added to each well. Plates were then incubated at 37 °C for 72 h. Cells were then fixed with 4% paraformaldehyde for 1 h at RT. The fixation solution and the semisolid overlay medium were then removed and cells stained with a crystal violet solution for 10 min. Thereafter, the stained cells were washed with PBS, plates dried, and plaque forming units (PFUs) counted.

### 2.11. Epitope Binning by Biolayer Interferometry

The panel of anti-stem mAbs was assessed for competitive binding using H1N1 CA/09 rHA on the BLI Octet RH16 system (Sartorius), as previously described [28]. Anti-Penta-HIS (HIS1K) biosensors (Sartorius) were first equilibrated in kinetics buffer (PBS + 0.5% BSA + 0.05% Tween-20) for 60 s to establish a baseline. Biosensors were then loaded with 100 μg/mL of CA/09 rHA protein in kinetics buffer for 60 s, followed by a 60 s baseline step in kinetics buffer. For competition analysis, biosensors were immersed in the first mAb (competitor) (100 μg/mL in kinetics buffer) for 300 s to allow association, then immersed in the second mAb (probe) (100 μg/mL in kinetics buffer) for an additional 300 s. Biosensors were regenerated through three cycles of alternating 0.1 M glycine, pH = 2.7, and PBS before proceeding to the next mAb competition set.

Competition levels were quantified as percent inhibition using the formula: percent inhibition = 100 × [(signal of probe mAb alone − signal of probe mAb with competitor mAb)/signal of probe mAb alone]. A percentage inhibition ≥ 75% was considered complete competition, 35–74% indicated moderate competition, and <35% indicated no competition.

### 2.12. PN-SIA49 Fab Generation

PN-SIA49 Fab was generated through immobilized papain-mediated cleavage of recombinantly produced IgG using the Pierce Fab Digestion Kit (cat. n. 44985, Thermo Fisher Scientific). The digestion reaction was allowed to proceed overnight at 37 °C with shaking. The following day, the digestion reaction was run on a HiTrap mAbSelect SuRe column (cat. n. 11003493, GE Healthcare, Chicago, IL, USA) and flow-through containing Fab was collected and buffer exchanged into 20 mM Tris, pH = 7.5, and 100 mM NaCl.

### 2.13. PN-SIA49 Fab:Y2 COBRA HA Complex Formation

PN-SIA49 Fab was added in a molar excess to Y2 COBRA HA in 20 mM Tris, pH = 7.5, and 100 mM NaCl buffer, and then incubated overnight at 4 °C to generate the PN-SIA49 Fab:Y2 COBRA HA complex. The mixture was run on a Superdex 200 Increase 10/300 GL column (cat. n. 17-5175-01, GE Healthcare) to isolate the Fab:HA complex from excess PN-SIA49 Fab. The complex was then used to prepare cryo-EM grids.

### 2.14. PN-SIA49 Fab:Y2 COBRA HA Cryo-EM Data Collection and Processing

PN-SIA49 Fab:Y2 COBRA HA complex, at a concentration of 1.58 mg/mL, was used to prepare grids. Data were collected on a Titan Krios at the Arizona State University Eyring Materials Center (Tempe, AZ, USA), using a magnification of 22,500 and defocus targets cycling between −0.80 and −2.60 mm.

In total, 6804 movies were imported into CryoSPARC, followed by motion correction and CTF estimation. The blob picker was used for initial particle picking to generate 2D class averages after particle extraction. Selected 2D class averages were then used for further particle picking using the template picker, followed by particle extraction, two additional rounds of 2D class averaging, and ab initio reconstruction. Afterwards, multiple rounds of local CTF refinement, global CTF refinement, and non-uniform refinement were performed with C3 symmetry imposed to generate a final map at 3.52 Å. This map was further postprocessed using DeepEMhancer version 0.14 [29] using the ‘highRes’ setting. Processing details are shown in Figure S1 and Table S1.

## 3. Results

### 3.1. PN-SIA49 and PN-SIA28 Binding Breadth

To further investigate mAb-HA interactions, binding kinetics were evaluated for each HA using biolayer interferometry. Both antibodies cross-reacted with all group 1 HAs, displaying high affinities (Kd < 50 nM), except for the binding of PN-SIA49 on Mich/15, Brisb/18. and Viet/04 (Kd < 100 nM). Remarkably, both mAbs showed strong binding to the four avian influenza strains tested (GyrFalc/14, Sich/14, ChickEgy/17, and TX/24).

**Figure 1:**
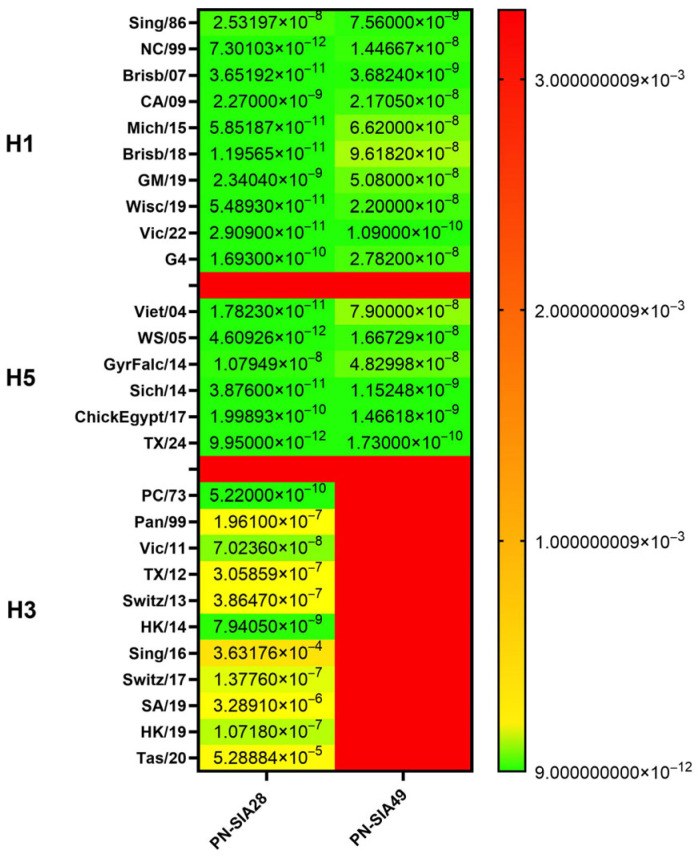
PN-SIA28 and PN-SIA49 binding kinetics heat map. PN-SIA28 and PN-SIA49 binding kinetics were measured using biolayer interferometry (BLI), and the equilibrium dissociation constant (Kd) (M) was determined against H1, H3, and H5 HAs for PN-SIA28, and H1 and H5 for PN-SIA49.

PN-SIA28 exhibited a Kd of 9.95 × 10^−12^ M, while PN-SIA49 showed a Kd of 1.73 × 10^−10^ M on TX/24 (Figure 1). To confirm these results, binding was also tested on cells infected with the Duck/22 H5N1 HPAIV strain (Figure S2).

As expected, for group 2 HAs, PN-SIA28 showed the strongest affinity for H3 PC/73 HA [16]. However, PN-SIA28 showed also a high affinity (Kd of 7.94 × 10^−9^ M) for HK/14 HA, and a slightly higher Kd for Vic/11 and HK/19 (Figure 1).

### 3.2. PN-SIA28 Neutralization Activity Against H3N2 HK/14

**Figure 2:**
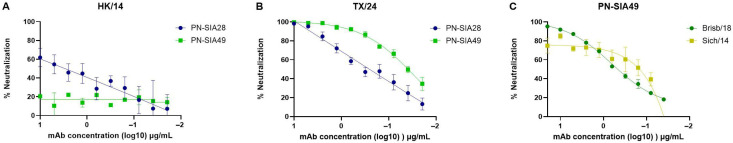
PN-SIA28 and PN-SIA49 neutralizing activity. (**A**) PN-SIA28 and PN-SIA49 neutralizing activity against the H3N2 HK/14. (**B**) PN-SIA28 and PN-SIA49 neutralizing activity against the avian strain TX/24 using a PV-based microneutralization assay. PN-SIA28 and PN-SIA49 were tested using a range concentration of 10–0.02 µg/mL (two-fold dilution). The neutralization was measured in RLU values that were normalized relative to the PV only and cells only. (**C**) PN-SIA49 neutralizing activity against Brisb/18 and Sich/14 using focus reduction assay (FRA). PN-SIA49 were tested using a range concentration of 20–0.078 μg/mL (two-fold dilution). All neutralization-antibody dose–response curves were modelled using a non-linear regression analysis.

A dose-dependent neutralizing activity was observed for PN-SIA28 against the H3N2 HK/14 strain (Figure 2) and with an estimated IC_50_ value above 5 μg/mL. No neutralizing activity was observed for PN-SIA49.

### 3.3. PN-SIA49 and PN-SIA28 Neutralization Activity Against H5 from Clade 2.3.4.4b

Both PN-SIA28 and PN-SIA49 showed a neutralizing activity with estimated IC_50_ values of 0.03 µg/mL for PN-SIA49 and 2.681 µg/mL for PN-SIA28 (pMN assay) (Figure 2B). The estimated IC_100_ values were 24 µg/mL for PN-SIA28 and 64 µg/mL for PN-SIA49 (MNT assay).

### 3.4. PN-SIA49 Neutralization Activity Against H1 and H5 Strains

We tested the neutralizing activity of PN-SIA49 against two viral strains belonging to different subtypes (H1 and H5). PN-SIA49 was observed to neutralize both strains, although with different levels of potency. As shown in Figure 2C, PN-SIA49 exhibited a stronger neutralizing activity against the Sich/14 strain, with an IC_50_ value of 1.518 × 10^−6^ µg/mL, and 0.6342 µg/mL for Brisb/18.

### 3.5. Prophylactic Activity of PN-SIA49

**Figure 3:**
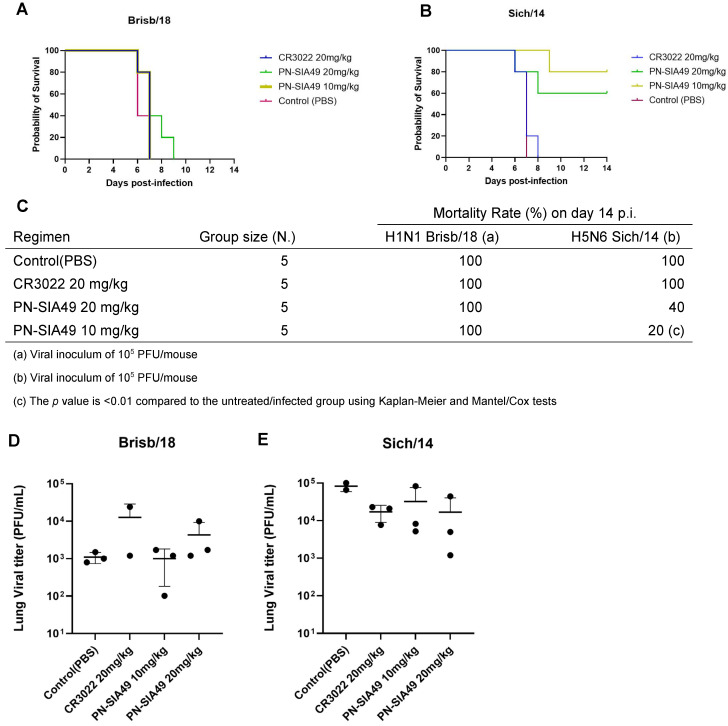
Prophylactic treatment with PN-SIA49. Effect of prophylactic administration of PN-SIA49 on the survival of mice intranasally infected with Brisb/18 (**A**) or Sich/14 (**B**) viral strains. Mice received 24 h before infection a single intraperitoneal dose of saline (PBS), CR3022 (IgG1, κ isotype control) at 20 mg/kg, and PN-SIA49 at 20 mg/kg and 10 mg/kg. (**C**) Summary of mortality rates and statistical analysis (Mantel/Cox) compared to the untreated/infected group. Lung viral titer of mice (n = 3/group) infected with Brisb/18 (**D**) and Sich/14 (**E**).

In the prophylaxis experiment, we observed a 100% mortality rate (MR) of mice infected with the H1N1 strain that received either the highest or the lowest dose of PN-SIA49 (Figure 3A,C).

This was corroborated by the marked body weight loss of the animals belonging to the same groups (Figure S3A). Moreover, we did not observe a statistically significant difference in the mean (±standard error of the mean [SEM]) LVT of mice infected with Brisb/18 that received 20 or 10 mg/kg of PN-SIA49 (4.3 ± 2.87 × 10^3^ PFU/mL; 1 ± 0.47 × 10^3^ PFU/mL) compared to untreated (PBS) mice (1.1 ± 0.208 × 10^3^ PFU/mL) (Figure 3D). On the contrary, we observed a different trend in mice infected with the H5N6 viral strain. A 60% survival rate was detected in the animals receiving 20 mg/kg of PN-SIA49, whereas the group that received 10 mg/kg of PN-SIA49 had a survival rate of 80%. The reduction of the mortality rate in this latest group of mice showed a statistically significant difference compared to the untreated and infected group (*p* < 0.01) (Figure 3B,C). The two groups of mice treated with PN-SIA49 displayed a significant body weight loss until 7 days p.i. and a rebound in the following days (Figure S3B).

Both groups of mice challenged with Sich/14 and treated with either 20 or 10 mg/kg showed a reduction in the LVT (1.67 ± 1.67 × 10^5^ PFU/mL; 3.2 ± 2.5 × 10^5^ PFU/mL) compared to the untreated group (8.3 ± 1.7 × 10^5^ PFU/mL); however, this difference was not statistically significant (Figure 3E).

### 3.6. Therapeutic Activity of PN-SIA49

**Figure 4:**
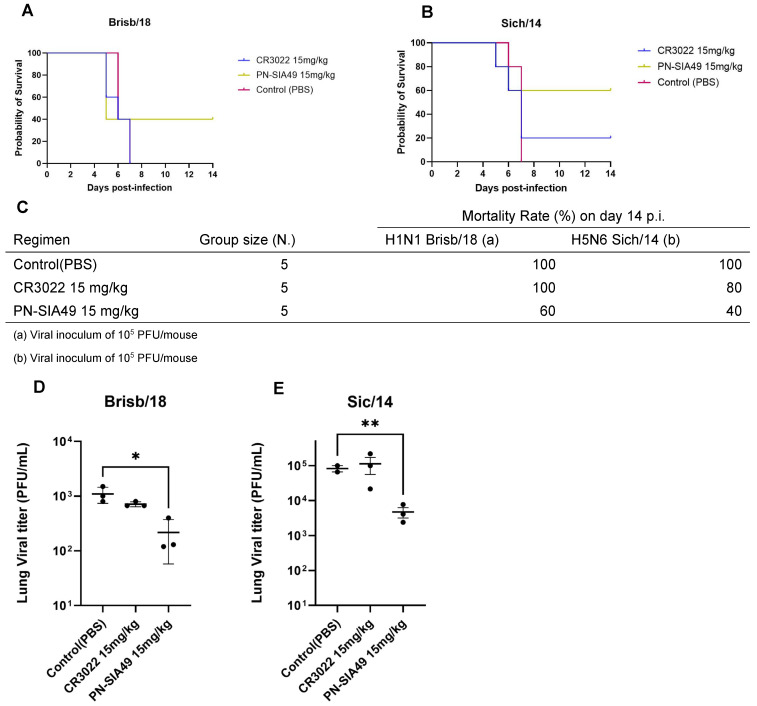
Therapeutic treatment with PN-SIA49. Effect of therapeutic treatment with PN-SIA49 on the survival of mice intranasally infected with Brisb/18 (**A**) or Sich/14 (**B**) viral strains. Mice received 24 h after infection a single intraperitoneal dose of saline (PBS), CR3022 (IgG1, κ isotype control) at 20 mg/kg and PN-SIA49 at 15 mg/kg. (**C**) Summary of mortality rates and statistical analysis (Mantel/Cox) compared to the untreated/infected group. Lung viral titer of mice (n = 3/group) infected with Brisb/18 (**D**) and Sich/14 (**E**). * *p* < 0.05, ** *p* < 0.01; *t*-test was used for all comparison to the untreated/infected group.

In the therapeutic in vivo experiment, we observed a reduction in the MR for mice treated with the 15 mg/kg regimen of PN-SIA49, when challenged with either the H1N1 or H5N6 viral strains. In particular, the survival rate for the mouse group infected with Brisb/18 was 40% (Figure 4A,C), whereas, for the mice infected with Sich/14, it was 60% (Figure 4B,C). Along with these results, PN-SIA49-treated mice infected with Brisb/18 showed a stabilization of body weight loss around day 7 post-infection, followed by a slight recovery starting from day 12 (Figure S3C). Meanwhile, PN-SIA49-treated mice infected with Sich/14 exhibited a steady increase in body weight beginning on day 7 post-infection (Figure S3D).

A significant difference between the mean (±standard error of the mean [SEM]) LVT of untreated mice and mice infected with Brisb/18 that received 15 mg/kg of PN-SIA49 (1.1 ± 0.2 × 10^3^ PFU/mL; 2.16 ± 0.9 × 10^2^ PFU/mL *p* < 0.05) was observed. Moreover, the same outcome was observed for mice challenged with Sich/14. Moreover, in this case, an approximate 1-log reduction in the LVT of mice treated with 15 mg/kg of PN-SIA49 (4.7 ± 1.56 × 10^3^ PFU/mL *p* < 0.01) (Figure 4C) was observed.

### 3.7. Competition Assay

**Figure 5:**
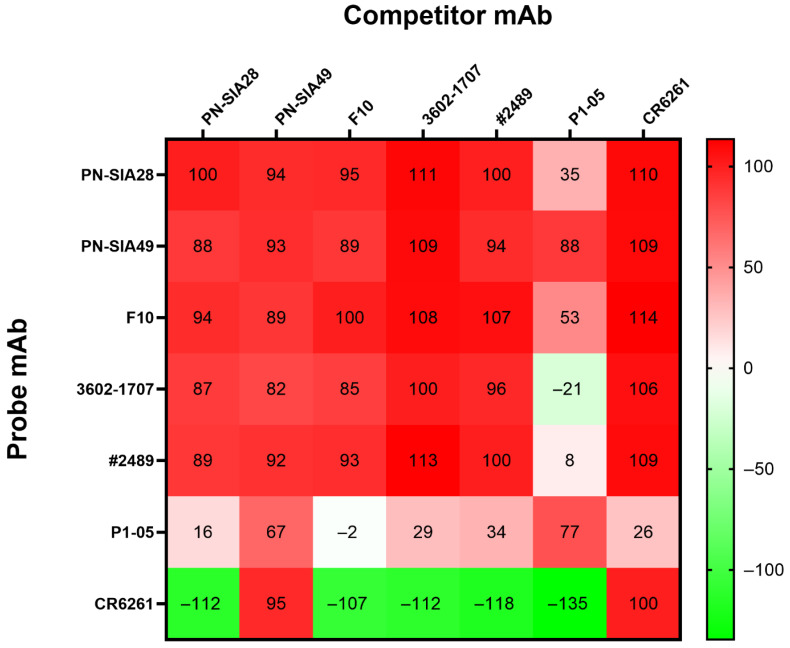
Epitope binning by biolayer interferometry. Competitive binding assays were performed by biolayer interferometry (BLI) using full-length CA/09 HA as a ligand and a set of distinct pairs of mAbs. Competition values are expressed as a percentage of the inhibition of binding of the probe mAb in presence of the competitor mAb. Red indicates complete competition, white moderate competition, and green no competition.

To map the regions bound by PN-SIA28 [16] and PN-SIA49 on H1 HA, reciprocal competition assays between these mAbs and several other HA stem-directed mAbs (F10 [30,31], P1-05 [32], CR6261 [33,34], 3602-1707 [35], and #2489 [36], were performed for assessing the possible overlap in their epitope recognition. As shown in Figure 5, PN-SIA28 and PN-SIA49 competed with all the HA stem-directed mAbs. However, while a strong reciprocal competition was observed among PN-SIA28, PN-SIA49, F10, 3602-1707, and #2489, an asymmetrical competition was observed between CR6261 when used as a competitor against all the other HA stem-directed mAbs.

Additionally, a moderate and reciprocal competition between PN-SIA49 and P1-05 was observed, suggesting that PN-SIA49 targets an epitope partially distinct from that recognized by the other HA stem-directed mAbs.

### 3.8. PN-SIA49 Fab Appears to Bind an Epitope Proximal to but Distinct from the HA Central Stem

**Figure 6:**
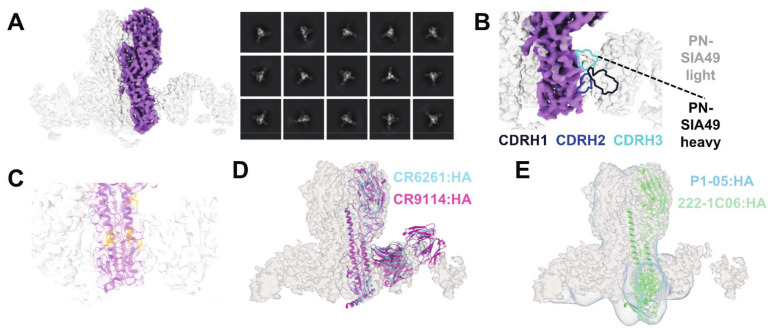
Cryo-EM analyses of the PN-SIA49 Fab:Y2 complex. (**A**) Electron potential map (**left**) and 2D class averages (**right**) of the PN-SIA49 Fab:Y2 complex. A monomer of Mich/15 HA, from PDB ID 7KNA, was fit into the map and colored in dark purple. (**B**) Densities of probable PN-SIA49 heavy chain CDRs from (**A**) at the Fab:HA interface. (**C**) The PN-SIA49 epitope from previous alanine scanning studies [15] mapped to the cryo-EM map-fitted HA from (**A**), with epitope residues colored in orange. (**D**) Comparisons of the binding orientations and epitopes of central stem-binding Fabs CR6261 and CR9114 to that of the PN-SIA49 Fab to Y2. CR6261 and CR9114 structures are from PDB IDs 3GBN and 4FQI, respectively. (**E**) Comparisons of the binding orientations and epitopes of anchor epitope-binding Fabs P1-05 and 222-1C06 to that of the PN-SIA49 Fab to Y2. The P1-05 Fab:Y2 map is from EMD-26586 and the 222-1C06 structure is derived from PDB ID 7T3D. The fitted structures and maps are labelled in their respective colors.

To structurally define the epitope of PN-SIA49, a cryo-EM was performed with the Fab fragment in a complex with the Y2 COBRA HA (Figure 6 and Figure S1).

This HA combines H1 HA sequences from 2014 to 2016 into a single antigen [37]. Therefore, this antigen is antigenically similar to recent post-2009 H1 strains, as has been observed previously based on studies of Y2-reactive antibodies from seasonally vaccinated human populations [32]. The 2D class averages and the electron potential map of the Fab:HA complex at 3.52 Å highlighted that the recognized epitope is on the stem domain of Y2 (Figure 6A). Although some orientation bias towards the top versus side views precluded model building, an analysis of the cryo-EM density after the fitting of the Mich/15 HA suggested that the PN-SIA49 Fab binds to its stem epitope exclusively using its heavy chain, based on the density of a long CDR loop with probable HA contacts (Figure 6B). This is likely contributed by its long 20-amino acid CDRH3. Moreover, the densities of the CDRH1 and CDRH2 loops that are likely to make contact can be observed. Previous studies have described the predicted binding site of PN-SIA49 based on alanine scanning [15]. The mapping of these residues onto the fitted HA revealed that they lay in close proximity to the putative heavy chain CDR loops of PN-SIA49 (Figure 6C). Moreover, consistent with the competition data, the PN-SIA49 Fab overlapped in its putative epitope with central stem mAbs CR6261 and CR9114 (Figure 6D). Some possible steric overlap between the PN-SIA49 and anchor antibodies P1-05 and 222-1C06 was also visible in structural comparisons, albeit to a lesser extent than for central antibodies (Figure 6E). Furthermore, considering that the competition assay uses full IgGs rather than Fabs, this may further explain the competition observed between anchor epitope antibodies and PN-SIA49 despite their epitopes being distinct from one another (Figure 5).

## 4. Discussion

Although seasonal influenza infections may receive less attention than pandemic threats, such as the COVID-19 pandemic, its cumulative impact on public health and the global economy remains substantial. Each year, millions of individuals are infected by the influenza virus, and, in the United States alone, the annual total economic burden to the healthcare system and society has been estimated at $11.2 billion [38]. In addition to this recurring burden, the potential consequences of future influenza pandemics must also be considered, given their capacity to cause severe global health crises and economic disruption.

Over the past century, there have been four influenza pandemics, three of which had a clear avian origin. In fact, influenza viruses, particularly the avian strains, have shown their ability to cause spillovers and, therefore, represent a continuous global health concern. The most recent example is represented by the current circulation of the avian influenza viruses belonging to the clade 2.3.4.4b. Although no cases of human-to-human transmission have been reported so far, this avian influenza strain has already caused numerous outbreaks in cattle and poultry, as well as several cases of animal-to-human transmission.

We previously described two human mAbs, PN-SIA28 and PN-SIA49, both cloned from a healthy individual with no known recent influenza virus exposure at the time of the sample collection, which neutralize the 2009 H1N1 pandemic influenza virus [14].

In this work, we expanded the characterization of these two antibodies, including the investigation of their biological activity against the current clade of avian influenza virus, to understand their possible role in managing future influenza outbreaks [39].

We focused the characterization of PN-SIA28 and PN-SIA49 on three of the most significant influenza viruses able to mediate human-to-human transmission and covering several decades, two belonging to group 1 H1 and H5 and one belonging to group 2 H3 HAs.

We demonstrated that these two mAbs recognize with different affinities a broad panel of H1 HAs and, more interestingly, also H5 HAs derived from avian influenza viruses, including strains belonging to the current circulating clade. Moreover, both antibodies showed a potent neutralizing activity against the currently circulating representative H5N1 strain (TX/24). Interestingly, the HA sequence of Duck/22 is identical to that of TX/24, so we can compare the two used neutralization systems, pMN and MN. Of note, the different IC values, obtained with pMN and MN, can be explained by the better accessibility of the HA epitope on the pseudotyped viral particles’ surface due to the absence of NA molecules, which primarily affects the binding of HA stem-directed antibodies, like PN-SIA28 and PN-SIA49 [40]. A second explanation might be the reduced density of HA molecules on pseudoparticles relative to the live virus favoring the mAb-mediated neutralization [41]. Furthermore, pMN assays against TX/24 showed a stronger neutralizing activity for PN-SIA49 compared to PN-SIA28, despite a higher affinity of PN-SIA28 against TX/24 HA compared to PN-SIA49. This discrepancy underlies that these two mAbs recognize different epitopes and that the accessibility of the epitopes may vary depending on the displayed HA glycoprotein as well as their role in the viral life cycle.

In this study, we describe for the first time the binding between PN-SIA28 and HA derived from an H3 strain circulating after 1985. The use of more sensitive methodologies and reagents (i.e., recombinant HA proteins) compared to those employed in previous studies [16,17] allowed the detection of a strong binding between PN-SIA28 and HA from HK/14. This finding was further explored by assessing the neutralizing activity of PN-SIA28 against the same strain. Interestingly, PN-SIA28 was able to neutralize the infection, although with a higher IC_50_ value compared to those measured against past strains. Future assays will be performed to validate the observed data and determine whether PN-SIA28 can also neutralize viral strains that, according to affinity measurements, show a weaker binding (Kd > 10^−8^). Furthermore, the assessment of the neutralizing activity of PN-SIA28 against phylogenetically related H3N2 strains to HK/14 will provide a more exhaustive profiling of the breadth of its biological activity.

Recently, the PN-SIA28-conferred protection of mice from an influenza strain belonging to the clade 2.3.4.4b has been described [9]. Here, we tested the PN-SIA49 for its ability to protect mice against the same clade. Importantly, PN-SIA49 was able to reduce the mortality and lung infection from the clade 2.3.4.4b H5N6 virus (Sich/14), in both prophylactic and therapeutic studies, even when tested at the lower dose (10 mg/kg in the prophylactic study). However, in both the prophylactic and therapeutic studies against the Brisb/18 strain, PN-SIA49 provided a lack of or lower protection than against the Sich/14 strain. This difference is likely due to the lower binding affinity of PN-SIA49 for Brisb/18 (Kd of ~10^−7^) compared to Sich/14 (Kd of ~10^−9^) (Figure 1).

We also further investigated the epitope of PN-SIA49 through cryo-EM and competition assays. In detail, cryo-EM confirmed that the PN-SIA49 Fab bound the stem domain of the H1 Y2 COBRA HA, consistent with previous observations of broad H1, H2, and H5 subtype neutralization and competition with the HA stem binding like mAb C179 [15]. Moreover, PN-SIA49 appeared to bind to an epitope proximal to, but distinct from, the HA central stem, a known target of several mAbs with broad subtype recognition, such as CR6261 [42] and CR9114 [43] (Figure 6D). Furthermore, unlike CR6261 and CR9114, PN-SIA49 binds to its epitope in an upward orientation as opposed to a downward orientation. Nevertheless, PN-SIA49 competes with CR6261 for the binding to an H1 HA (CA/09). All the differences outlined above and including the relative contributions of the heavy and light chains as well as the binding affinity can contribute to the difference in in vitro neutralization and in vivo protection profiles shown by PN-SIA49 [9,44].

It is noteworthy that, similarly to other mAbs targeting the HA central stem [45], PN-SIA49 is also endowed with antibody-dependent cellular cytotoxicity (ADCC) [20], as recently reported, and this Fc-mediated activity may further contribute to the PN-SIA49 in vivo conferred protection [46].

The epitope of PN-SIA49 did not overlap with the anchor epitope bound by previously characterized mAbs, such as 222-1C06 [47] and P1-05 [32], with no overlap of the PN-SIA49 Fab density maps with these mAbs (Figure 6E). However, the proximity of these epitopes could explain its partial competition observed with these mAbs. These data show that PN-SIA49 binds a stem epitope on HA, close to the epitope conserved across several HA group 1 subtypes, contributing to its broad neutralization and HA binding profile [48].

## 5. Conclusions

This work demonstrates that mAbs derived from subjects with no known history of exposure to currently circulating influenza virus strains, including pre-pandemic strains, are capable of broad and extended neutralization and protection. These findings highlight that the elicited humoral response can include antibodies with extraordinarily broad reactivity and reveal the presence of a conserved region on HA shared across a broad panel of influenza viruses within the same group (PN-SIA49) or across different groups (PN-SIA28).

Importantly, these antibodies are part of the memory B cell antibody repertoire that can be potentially recalled upon infection or influenza seasonal or ad hoc vaccination in the event of a pandemic outbreak.

The biological and structural characterization of conserved HA epitopes is pivotal for the design of next-generation influenza virus vaccines aimed at eliciting/recalling a broadly neutralizing and protective antibody response. Importantly, here, we showed that these antibodies are able to bind next-generation influenza vaccine antigens, like COBRA HA, that will soon enter clinical trials, highlighting the capability of these antigens to potentially recall antibodies possessing these epitope specificities and broad biological activity.

## Data Availability

The original contributions presented in this study are included in the article/Supplementary Materials. Further inquiries can be directed at the corresponding authors.

## Supplementary Materials

The following supporting information can be downloaded at: https://www.mdpi.com/article/10.3390/vaccines14010071/s1, Figure S1: Cryo-EM processing, map resolution, and particle distributions. Figure S2: PN-SIA28 and PN-SIA49 binding on H5 infected monolayer. Figure S3: Weight loss of infected mice following either a prophylactic or therapeutic regimen with PN-SIA49. Table S1: PN-SIA49 Fab:Y2 cryo-EM data collection and refinement statistics.
