# Supplementary material for: Extended Heterosubtypic Neutralization and Preclinical Model In Vivo Protection from Clade 2.3.4.4b H5 Influenza Virus Infection by Broadly Neutralizing Antibodies
Source: Vaccines (Basel). 2026 Jan 8;14(1):71. doi: 10.3390/vaccines14010071 (PMC12846329; doi:10.3390/vaccines14010071)
Supplement: Supplementary file 1 [file vaccines-14-00071-s001.zip › vaccines-4041089-supplementary.pdf]

Supplementary Figures

Figure S1

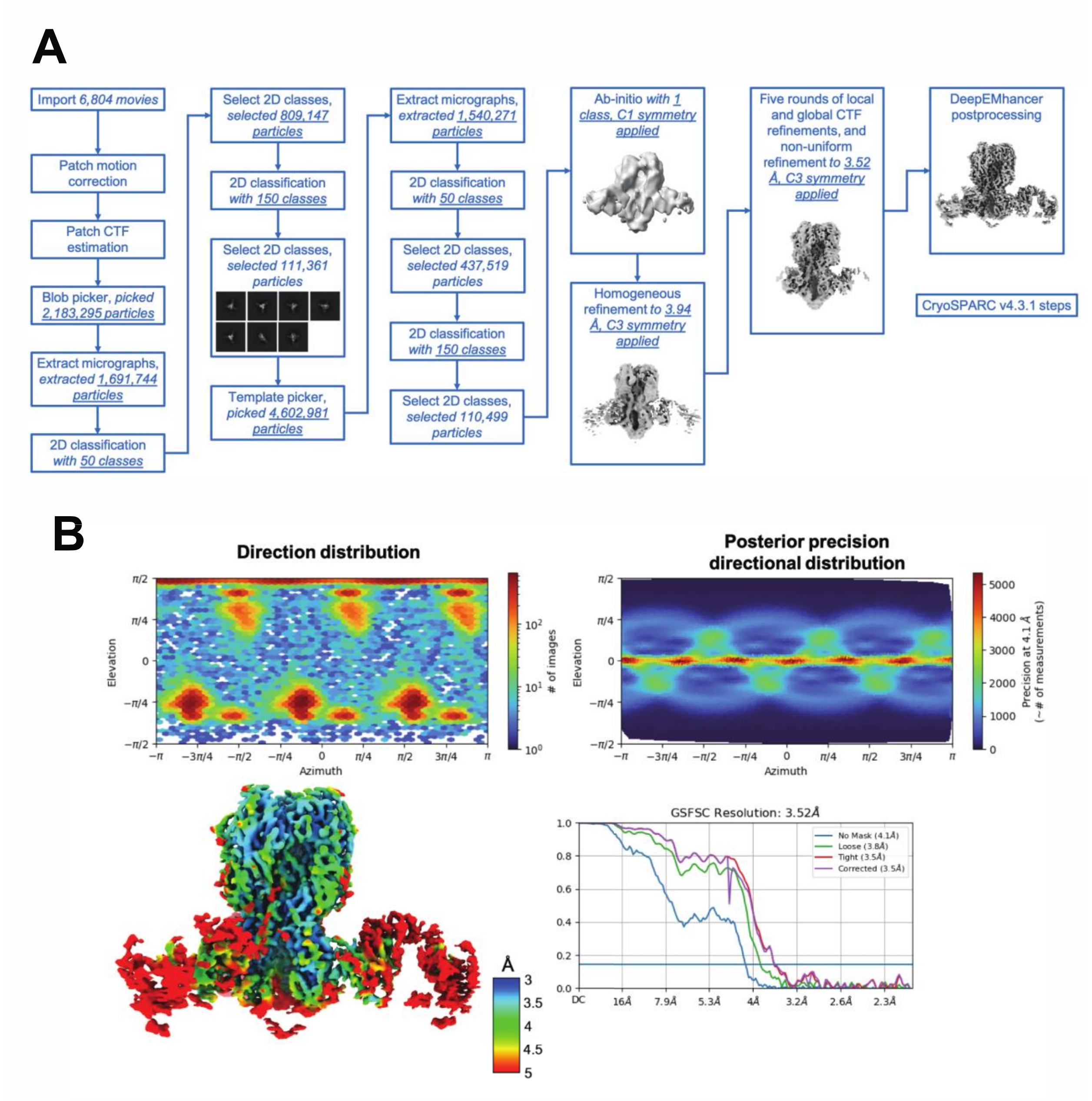

Figure S1. Cryo-EM processing, map resolution, and particle distributions. (A) Cryo-EM processing workflow. (B) Particle orientation distributions (top) and the local resolution map of the final map with the gold-standard FSC resolution (bottom).

Figure S2

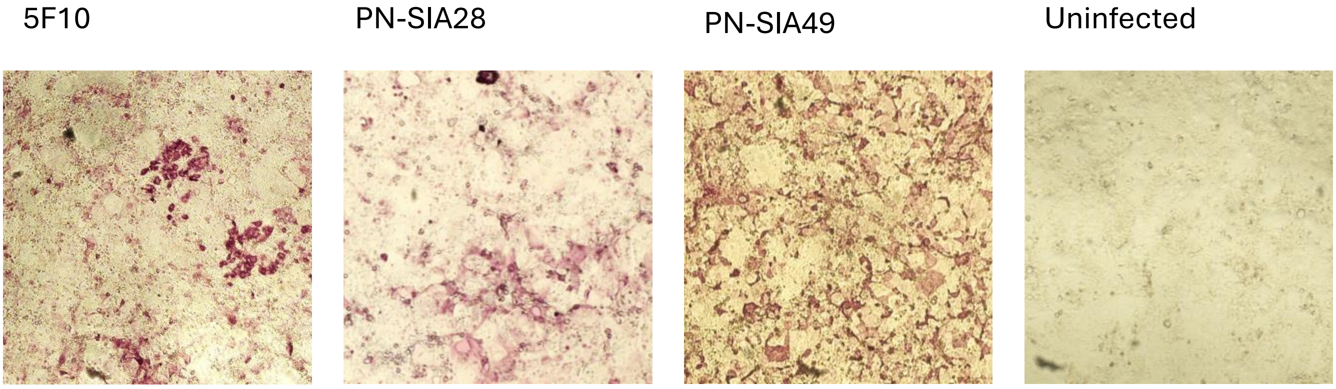

Figure S2: PN-SIA28 and PN-SIA49 binding on H5 infected monolayer. PN-SIA28 and PN-SIA49 were tested on MDCK cells infected with 100 $TCID_{50}$ of A/duck/Italy/326224/2/22VIR909/2022 H5N1 HPAIV for their binding. mAb 5F10 was used as positive control of the infection while uninfected monolayer as negative control. The binding was observed under an optical microscope at 10x magnification.

Figure S3

A

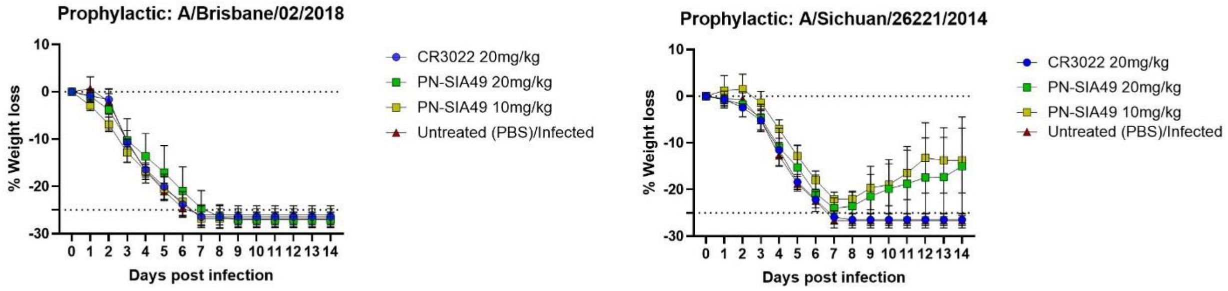

B

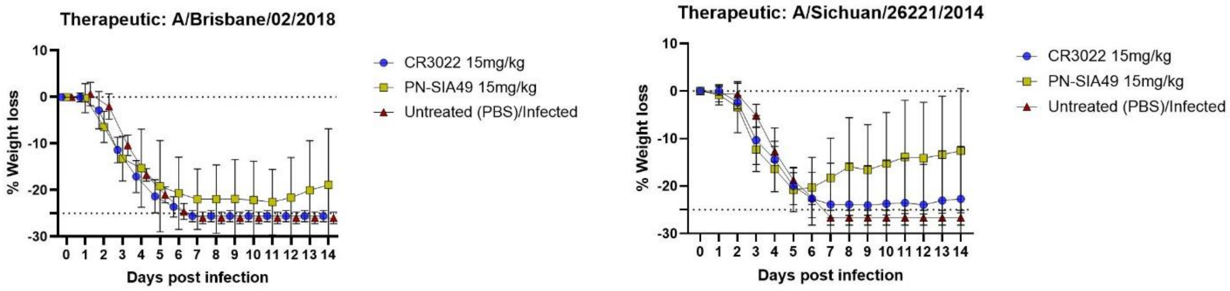

Figure S3. Weight loss of infected mice following either a prophylactic or therapeutic regimen with PN-SIA49. (A) Mice infected with either Bris/18 or Sich/24 undergoing PN-SIA49 prophylactic regimen. (B) Mice infected with either Bris/18 or Sich/24 undergoing PN-SIA49 therapeutic regimen. Mice that lost more than 25% of body weight were suppressed.

Supplemental tables

Table S1. PN-SIA49 Fab:Y2 cryo-EM data collection and refinement statistics.

PN-SIA49 Fab complexed with Y2	
EMDB ID	EMD-45424
**Data collection**	
Microscope	Titan Krios
Detector	K2 Summit
Voltage (kV)	300
Magnification	22,500
Defocus range (µm)	-0.80 to -2.6
Pixel size (Å)	1.03
Number of frames	40
Nominal dose	57.96 e/Å^2
No. of images collected	6,804
**Reconstruction**	
Software	CryoSPARC
Number of used particles	110,499
Symmetry	C3
Final resolution (Å) ($FSC_{0.143}$)	3.52 Å
Postprocessing software	DeepEMhancer[1]
